# Substance misuse in later life: challenges for primary care: a review of policy and evidence

**DOI:** 10.1017/S1463423618000440

**Published:** 2019-06-20

**Authors:** Rahul Rao, Ilana Crome, Peter Crome, Steve Iliffe

**Affiliations:** 1 Consultant Old Age Psychiatrist, South London and Maudsley NHS Foundation Trust, London, UK; 2 Visiting Researcher, Institute of Psychiatry, London, UK; 3 Emeritus Professor, Keele University; 4 Honorary Professor, Queen Mary University, London, UK; 5 Visiting Professor, St George’s University, London, UK; 6 Senior Research Fellow, Imperial College London, London, UK; 7 Emeritus Professor, Department of Primary care and Population Health, University College London, London, UK; 8 Emeritus Professor of Primary for Older People, University College London, London, UK

**Keywords:** addictions, geriatrics, older people, primary care

## Abstract

**Background:**

Substance misuse in older people represents a growing clinical and public health problem within primary care.

**Aim:**

The aim of article is to explore policy and research evidence for informing best practice in the assessment, treatment effectiveness, treatment implementation and approaches to recovery for older people with substance misuse in primary care.

**Methods:**

Relevant search terms were used to examine the databases MEDLINE, EMBASE, CINAHL and PsychINFO up to January 2016.

**Results:**

An age-sensitive approach is required to overcome barriers to assessment. Training is essential for developing relevant knowledge, skills and attitudes. Clinical audit be used to develop care pathways, particularly for older people with dual diagnosis. There is also a need to develop closer links between primary care and the secondary care specialties, as well as added value in working with carers and voluntary agencies.

**Discussion:**

Further research is needed to inform more effective approaches to treatment. Adequate funding for workforce development and quality improvement in service development are also essential to improve health outcomes and quality of life in older people with substance misuse.

## Introduction

The number of older people in both the United Kingdom and in other developed countries has risen considerably over the past 20 years. This has been accompanied by a disproportionate rise in the number of older people who use substances, particularly alcohol (Rao *et al*., 2015a). This is likely to continue for at least the next two decades (Institute of Alcohol Studies, 2013). The rate of alcohol related deaths in the United Kingdom is highest in the 55–74 age group (Office of National Statistics, 2015), with the number of older illicit substance misusers likely to double by 2020 (Han *et al*., 2009). The rise in substance misuse in older people is also associated with inappropriate prescribing, drug interactions, the use of over the counter medicines and substances purchased on the internet. Such misuse is associated with both mortality and long-term ill-health.

The main purpose of this paper is to critically review both recent policy and the evidence underpinning clinical practice in older people with substance misuse. The main aims are to use the policy review to inform future practice and to use the evidence base to better inform clinical practice in the assessment, detection, treatment and recovery of older people with substance misuse in primary care.

The methodology used in this paper used the search terms ‘policy’; ‘practice’, ‘assessment’, ‘treatment’ and ‘recovery’ using the databases MEDLINE, EMBASE, CINAHL and PsychINFO up to January 2016.

## Background

Older people with substance misuse present with multiple and complex needs. Because of their co-morbidities, they are high users of primary care services, as well as of emergency medicine and trauma, addiction services, old age psychiatry and geriatric medicine services. However, the nature and extent of the problem has been unclear, given that integrated care for this population is lacking. The pressing need to examine the whole area in greater detail, both in the context of public health and clinical practice, resulted in the first report from the Royal College of Psychiatrists Substance Misuse in Older People report, Our Invisible Addicts (Crome *et al*., 2011).

Our Invisible Addicts served to drive forward key areas for further examination at several levels. At the level of service development, this involved producing clinical guidelines on care pathways, exploring the evidence base for safe drinking limits and developing training packages for health professionals.

There is also a need to remove barriers to assessment and treatment, exploring drug treatment interventions, improving the knowledge base for effective treatments from epidemiological research and exploring barriers to service provision from clinical audit. At an ethical level, it is also necessary develop, implement and promote service delivery based on need, in an age-appropriate way via multi-agency partnership

It was surprising to find that about one in five men over the age of 65 years and one in 10 women over the age of 65 are drinking above ‘safe recommended limits’ for adults (NHS Information Centre, 2012), with a more recent study finding an overall prevalence of one in five for both men and women combined (Rao *et al*., 2015b).

There has been considerable discussion as to what constitutes a ‘safe limit’ for older people, following publication of the report (Crome *et al*., 2012). The report also highlighted that although the rate of smoking has shown a decline over time, about one in 10 people over the age of 60 are still smokers (NHS Digital, 2015). This contrasts with the number of people over the age of 40 who are entering treatment for illicit drug misuse, which is rising (Benyon *et al*., 2010).

Several other key messages emerged from Our Invisible Addicts. Older people often show complex patterns of mental and physical health presentations which may be atypical, subtle or non-specific and may well be missed. Iatrogenic complications may arise from inadvertent use of prescription and over the counter medications that interact with each other and with substances, even at low doses.

A combination of stigma, prejudice, lack of training and therapeutic nihilism can mean that substance use in older people is not fully assessed and therefore not treated. The comprehensive assessment undertaken by clinicians aware of the special needs of older people is a prerequisite for safe and effective care of the older substance misuser.

This may mean that every general practitioner undertakes a screen for substance use in people over the age of 65. There is also a need for vigilance by primary care practitioners over non-specific clinical presentations where substance use may be overlooked. Substance misuse is associated with a very wide range of conditions including hypertension, liver dysfunction, diabetes mellitus, falls, delirium, cognitive impairment and decline, depression, anxiety, self-harm and suicide.

Our Invisible Addicts noted the limited evidence base for treatment outcomes (Moy *et al*., 2011), which has since been updated with a further systematic review showing that older people do as well, if not better, than their younger counterparts in improving health outcomes (Rao, 2013; Bhatia *et al*., 2015).

## Moving from policy to practice

Our Invisible Addicts laid a firm foundation for the development of a comprehensive clinical guide for the recommendation of best practice for a range of problems associated with substance misuse in older people. This later came in the form of an Information Guide (Rao *et al*., 2015a) to assist clinician decision making and improve health and social outcomes. It was developed over three years by experts working across health, social care and the voluntary sector. The guide was primarily for health and social care professionals, but was also intended to inform commissioners, researchers, educators, policy makers and the voluntary/private sector.

The main objectives were the prevention of premature death in older people with substance misuse and enhancing quality of life in long-term conditions (including dual diagnosis).

During development of the Information Guide, other guidance had been developed for substance misuse, which had a bearing on older people. The first of these was services for the identification and treatment of hazardous drinking, harmful drinking and alcohol dependence in children, young people and adults, published by the National Institute for Health and Clinical Excellence (NICE) (2011).

The NICE guide noted the need for lower thresholds for high-risk groups (including older people) when commissioning inpatient and residential medically assisted alcohol withdrawal services. It was supplemented by examples of good practice relevant to primary care that included community-based interventions, addressing complex needs, equality of access to services and patient experience.

The 2015 Information Guide was also influenced by the need to develop partnerships with public health, with the publication of a NICE public health briefing (NICE, 2012). It marked a turning point in the commissioning of prevention and treatment interventions for substance misuse.

As of April 2013, public health functions and those functions previously held by the National Treatment Agency were formally transferred to Local Authority Health and Wellbeing Boards. The Information Guide had several other aims.

It made recommendations for good practice over a wide range of problems within assessment, treatment and care; it assisted clinician decision making, with specific attention to the physical and psychosocial aspects of ageing, and sought to improve health and social outcomes, reduce harm from substance misuse and to promote recovery and community integration through the provision of high-quality care. There was a focus on clinical encounters of varying complexity, based on problems and settings rather than on disorders alone. It used vignettes, algorithms for care pathways and signposting to best practice, together with identifying individual and organisational barriers, so that ways to overcome these could be formulated.

The first section of the Guide is relevant to primary care and covered general approaches, including initial assessment; psychosocial interventions; family and carer support; legal and ethical aspects, as well as case management and care planning. In the second chapter, a more specialised approach was taken, focussing on emergency physical and psychiatric presentations; withdrawal syndromes; community management of the long-term effects and maintenance treatment of substance misuse; alcohol related brain injury; the recovery model; older women and alcohol problems; drug interactions; and driving and substance misuse.

## Key components in assessing older substance misusers

Barriers to assessment can be considerable and include ageism, collusion, under-reporting and lack training by health professionals. Once these barriers are recognised and overcome, it is important to be vigilant over risks like recent bereavement, as well as retirement, social isolation and immobility.

Gathering collateral information is invaluable, with a variety of possible sources such as carers (taking account of information sharing and confidentiality); general practitioner and community nurses; hospital discharge summaries; social care assessments; home carer and day-care reports.

Asking about the ‘geriatric giants’ of immobility, instability and falls, incontinence and impairment of intellect is essential in general practice and community nursing, as is taking a nutritional history. Allowing adequate time and paying heed to problems with sight, hearing, and language deficits is imperative. On examination, attention should be paid to general frailty, mobility, skin breakdown, pain on movement and constipation.

Assessing functional status through activities of daily living can also give clues as to the risks associated with substance misuse, such as tremor and falls. An alternative way to take a functional history is to ask patients to describe a typical day or week and establish where they and their relatives or carers perceive or experience difficulties. Enquiring into medication management and exploring potential drug interactions is essential. Psychiatric diagnoses associated with substance misuse include depressive disorder, anxiety-related disorders, alcohol-related brain injury and psychotic disorders.

Substance use in older people is often associated with ethical dilemmas around capacity, particularly when there is conflict between capacity and the role of practitioner in encouraging the older person to give up substance misuse. This is especially relevant because one of the core features of dependence syndromes is the persistence of substance misuse despite awareness of the harm from the substance being taken. Using the core feature of harm awareness, an assessment of mental capacity in substance misuse may therefore help to distinguish unwise decisions from a lack of mental capacity *per se*.

Another legal aspect relevant to older people is elder abuse. Substance abuse is more likely to occur in the perpetrators of the abuse compared with the person suffering abuse (Anetzberger, 2005).

In those perpetrators with a health problem, heavy consumption of alcohol or drug substances is not uncommon; this may even extend to care settings, where theft from care staff to fund substance misuse may occur. Women with a neurological or mental disorder and abuse drugs or alcohol are at highest risk of elder abuse (Friedman *et al*., 2011).

The most challenging area of assessment in older people with substance misuse is those with dual diagnosis at risk of self harm and those with alcohol-related brain damage (ARBD). In both cases, as well as an assessment that considers age-specific problems and risks, there should also be a full risk assessment carried out that includes the documentation of safeguarding, mental capacity, care planning and use of the care programme approach (CPA). The CPA should also include both crisis and contingency plans that are seamless in their interface with other agencies such as general practice and community nursing, substance misuse services, old age psychiatry, geriatrics, emergency, social and housing services.

## Treatment effectiveness and implementation of age-sensitive treatment

It is now well established that not only is the number of older people in our communities increasing, but also that their use of substances, particularly alcohol and prescription drugs, is rising. This is reflected in their care needs: older people are more likely to require hospital admission due to multiple problems, and this makes care costly.

North American studies indicate that the substance problem for which elders most often seek treatment is alcohol. It appears that only 6–7% of high risk drinkers over 60 years old are receiving the treatment that they require. Older drinkers are less likely to declare that they have a problem, more likely to have low dependence on alcohol, less likely to demonstrate hostility and more likely to be motivated to abstain.

The service delivery system is unprepared, partly because we are dealing with an invisible epidemic. This is due to numerous factors such as ageism, denial, stereotyping, non-specific symptoms, complex multiple diagnoses; in addition to the stigma, shame and isolation which older substance users experience. However, since older people are more likely to be in contact with primary care, there is significant potential to identify problems associated with alcohol misuse.

There are gaps in information about treatment prevalence, awareness by older people of their treatment needs and provision, the facilitators and barriers to treatment, the risk and protective factors in early and late onset substance misusers, and the influence of comorbid conditions.

However, there is sufficient information on treatment effectiveness in substance misusing adults, and an increasing body of work on older people, to justify ensuring that older people can access treatment interventions and options. To understand how treatment may beneficial, we need know who comes for treatment, what they expect from treatment, the healthcare framework and setting in which treatment takes place, what specific interventions are administered, and, of course, the outcome of treatment.

Two recent reviews of psychosocial treatment have been conducted which indicated that most studies were carried out in developed countries (Moy *et al*., 2011; Bhatia *et al*., 2015). Interventions included psychosocial approaches and pharmacological treatments for alcohol consumption, prescription drug misuse, cigarette smoking and illicit drugs, and were delivered in a range of healthcare settings, including primary care. It is obvious that more research needs to be done, but nonetheless, the key message is that older people should not be barred from treatment because of age.

In the research that has been undertaken in this group, consistently positive findings emerge from studies on psychosocial treatment for substance problems in the older patient. Taken overall, the studies demonstrate that older people do want to abstain; have the capacity to change; can be successfully offered help by physicians; respond well to brief advice and motivational enhancement therapy; can be treated outside an age-specific programme; can achieve improvement in outcomes across the range of domains (mental and physical health, relationships, legal, occupational and financial issues) at least comparable to younger adult populations, and perhaps even better; and have the prospect of long-term recovery.

Two recent studies on alcohol misuse in older people have been undertaken (Schonfeld *et al*., 2010; Moore *et al*., 2011). These are the BRITE (Brief intervention and treatment for elders) and HLAYA (Healthy living as you age) studies that follow on from others undertaken previously (Fleming *et al*. 1999; Bartels *et al*., 2004), namely GOAL (Guiding older adult lifestyles) and PRISM-E (Primary Care Research in Substance Abuse and Mental Health for the Elderly) further elucidate the value of intervention.

For example, BRITE reported a reduction of alcohol use and problems from 80 to 18%, though it is difficult to draw definite conclusions due to the variability of the interventions and because it was not controlled (Schonfeld *et al*., 2010). The HYALA study demonstrated that there was improvement in both the controlled condition (advice) and intervention (integrated care) groups at 12 months (Moore *et al*., 2011).

This is in keeping with the PRISM-E study which found that patients did better in integrated mental health and substance misuse care in primary care compared with referral to specialist providers.

There is also compelling evidence regarding tobacco cessation (Bhatia *et al*., 2015). Older people are less likely to be prescribed treatments even though quit rates are higher than in a younger age groups. Cognitive behavioural therapies and combined brief intervention, telephone calls and nicotine replacement treatments, were shown to be effective. For prescription medication misuse, counselling has been shown to be effective.

There are a few small pharmacological studies on older alcohol misusers, and those that have been undertaken indicate that drugs such as acamprosate, disulfiram and naltrexone are effective, safe and well tolerated. Medications can be cautiously administered in older people by experts experienced in the fields of addiction and geriatric medicine and monitored assiduously. There should be a lower threshold for type of medication (eg, benzodiazepines should be short acting to prevent accumulation and sedation), and for inpatient admission (for withdrawal due to medical complications). Any pharmacological intervention needs to be offered in the context of psychosocial treatments.

## Distinctive issues for/about older substance misusers

There is always a dilemma in discussing health issues in older people whether one should concentrate on what is different from younger people or what is similar. The important point is that older people cannot be considered as a homogenous group and some older substance users will have all the same features as people in mid-adult life whilst the clinical features in others will be very much influenced by the popular (mis)conceptions of old age: multiple physical and mental health morbidity, functional decline, relative poverty and social isolation. For example, 60% of people aged 60–64 have one or more co-morbidities but that means that 40% do not.

On the other hand, in the very old (over 90s), over 90% have a co-morbidity with a quarter having five or more conditions (Melzer *et al*., 2015). Age UK (2015) have produced a useful factsheet summarising these differences.

Such differences when they occur may be factors in the aetiology of substance misuse, the ways in which substance misuse may present and in how treatment may have to be adapted to be optimally effective.

The assessment of older substance misusers should be based on the principles of comprehensive assessment. Whilst in hospital this will usually be done by different professional groups contemporaneously, this may require a more sequential approach in the community, with a full picture being only available by a combination of clinic and home visits. The presence of frailty (most commonly defined as a combination of tiredness, weakness, low physical activity, weight loss and slow walking speed) should be assessed. Frail patients are more likely to fall or require hospitalisation and substances are likely to increase risk.

A recent US community study found a prevalence rate of frailty for over 65s of 15% (Bandeen-Roche *et al*., 2015). Cognition will also need to be assessed using, for example, the General Practitioner Assessment of Cognition (http://gpcog.com.au).

There is an obvious association between multi-morbidity and multi-prescribing, which in turn leads to increased accessibility and the potential for drug sharing. This is compounded by the guideline-suggested use of multiple drugs for the same condition, for example, for diabetes, hypertension and ischaemic heart disease.

Age related decline in renal function will cause drug accumulation and greater effects in drugs excreted by that route and the metabolism of many drugs which are metabolised in the liver is impaired particularly in the frail population. Older people are, as a rule, more sensitive to the effects of drugs that act on the nervous system and are thus more likely cause adverse events such as falls. Drugs with sedative effects and especially those with anti-cholinergic effects may cause confusion and increase the effects of substances. As pain is also a feature of ageing, opioid drugs with abuse potential are likely to be prescribed if symptoms do not abate with milder analgesics. Drug–drug interactions are much more likely.

Importantly services for older people may need to be adapted their distinctive needs. The physical ability to travel to treatment and support venues, sensory loss (eyesight and hearing) and concomitant physical health problems (eg, incontinence) may all be barriers for which individual solutions may be required.

## The presentation of substance misuse in older people

In older people – especially women – substance misuse is a complex, dynamic phenomenon shaped by experiences including violence, mental health disorders and social obligations such as caring for others (Koenig and Crisp, 2008). It may also be overshadowed by other changes in health and functional ability, making it a biopsychosocial problem typical of general practice.

This complexity means that substance misuse in older people is often not recognised, or recognised but poorly treated (Blow and Barry, 2012). Many barriers encountered by the clinician (time, knowledge about the patient and their medical history) and created by the patient (denial, communication problems, discomfort realising or admitting and discussing the problem) need to be overcome to engage with substance abuse in older people. Symptoms of substance misuse, including cognitive impairment and falls, can be easily confused with other conditions of ageing.

Recognition of substance abuse can be challenging, but the impact of substance abuse on older people justifies attempts at early recognition. For example, there is good reason to be concerned about alcohol misuse in older people with depression. Drinking more than 21 units of alcohol per week is positively associated with symptoms of depression and/or anxiety, as well as with perceived poor physical health (Kirchner *et al*., 2007).

It was once believed that illicit drug users ‘matured’ out of their drug use, but there is evidence from the United States that, for example, older heroin users do not reduce their use as they age (Rosen *et al*., 2011). The population of older methadone users is also increasing (Rosen *et al*., 2008) and their quality of life is poor (Rajaratnam *et al*., 2009).

Primary care practitioners may face rising demand for medical care from this population because the ageing process appears to be stimulated by long-term opiate use (Reece, 2010), with rapid physiological ageing promoting multi-system disease (Reece, 2012).

Older people with a history of heroin dependence have poorer physical health and social functioning than their non-dependent peers (Grella and Lovinger, 2012), and show high levels of major depression, post-traumatic stress disorder, generalised anxiety disorder, arthritis and hypertension (Rosen *et al*., 2008).

There is evidence even in abstinent heroin users of damage to the prefrontal cortex, affecting executive functioning, memory and attention control (Cheng *et al*., 2013). Enquiry about past and present opiate use in ageing ‘baby boomers’ with major disorders should become routine in primary care, and in memory assessment pathways.

Misuse of long-acting benzodiazepines is associated with multiple risks, including falls, road traffic accidents, drowsiness and ataxia, confusion, and impaired psychomotor function. Dependence can lead to anxiety, depression and cognitive impairment causing further morbidity in this already vulnerable population.

Those who misuse psychoactive prescription medicines are not easy to identify (Folkman *et al*., 1987). They do not have more stresses than non-users and do not cope with them differently, but they experience the stresses more intensely, feel more threatened by them and are more dissatisfied with their own coping abilities. Primary care practitioners need to develop a low threshold of suspicion for substance misuse when working with older patients with multiple symptoms and disorders.

## Implementation of treatment relevant to primary care

Since there is no single empirically supported psychosocial intervention that is superior, primary care practitioners need to be responsive to the needs of older people and to support adaptive coping strategies. The techniques which are likely to yield benefit – as in younger people – include brief interventions, motivational interviewing, motivational enhancement and cognitive behavioural therapy.

The components of age-sensitive treatment include a biopsychosocial assessment, treatment plans and goals, and regular re-assessment. Comorbid issues such as pain, cognitive impairment and depression are some of the most common co-occurring conditions which influence treatment outcome so protocols for referral to addiction and geriatric services are fundamental to care coordination of psychosocial and pharmacological interventions.

Staff need to be well trained, to enjoy working with older people, to be flexible to change with the fluctuating needs of their clients, as well as being cognisant of the appropriate goals, approaches, location, mode and duration of treatment for older people.

Other problems which may affect older people and are at least as important as the substance use, need to be given due recognition, such as accommodation, finance, physical illness, cultural differences, transport and accessibility. Patients may need to work at a slower pace, with shorter treatment sessions, and with the opportunity for reviewing and summarising information in a written format. Other components may include mutual self-help, age segregated treatment, care coordination and the use of information technology which may be less stigmatising.

Older age should not be a barrier to treatment. Older people have been reported to respond to interventions such as brief interventions, multi-component interventions, educational interventions and counselling. Older people can be offered treatments that are of proven effectiveness in the adult population, and that can be adapted to the needs of older people but should be monitored by a multi-disciplinary team including specific expertise in addiction and ageing. All substance use should be considered: alcohol, tobacco, polypharmacy, illicit drugs, over the counter medication and misuse of prescription drugs.

In the future, older people need to be enrolled in trials of pharmacological agents and psychosocial interventions. Combinations of treatments need further study so that findings can inform improved decision making or understanding the mechanism of action. Longer-term study may lead to recommendations for the utilisation of specific interventions and the development of appropriate age-sensitive treatment programmes so that service models can evolve collaboratively with a choice of input.

Studies in non-western settings are needed so that the contribution of culture, availability of resources and types of treatment setting can be considered. Since most studies were undertaken in primary care, there is further potential for enhancement of detection and treatment in primary care as well as integration with specialist mental health services since there is a high degree of comorbidity. This is cause for optimism.

We acknowledge that we still need to assess and tailor treatment systems that are more fitting for older substance misusers. However, much can be delivered in primary care supported by specialist addiction, geriatric and mental health services, as well of course, medical and social services.

## Approaches to recovery

Recovery from substance misuse in older people requires an integrated approach to care that considers both the interface with other agencies and using knowledge, skills and attitudes that tailor the recovery plan at an individual level using a ‘biopsychosocial’ approach. Such care involves shared decision with disciplines such as addiction psychiatrists, old age psychiatrists, mental health nurses, psychologists, social workers, occupational therapists and support workers. Each team member has a unique perspective around medical, cognitive/behavioural, social and functional aspects of lifestyle and illness.

In the United Kingdom, the development of an integrated service model has been developed in an area with high rates of alcohol misuse (Rao and Shanks, 2011). The strategy involves key performance indicators involving training in core skills in substance misuse for frontline clinical staff, audit of drug use and misuse. This includes cigarettes, illicit, prescribed and over the counter medication. It also includes alcohol screening from electronic patient records and identifying ‘champions’ within each clinical multi-disciplinary team who undergo advanced substance misuse training and offer supervision to clinical staff. There has also been considerable progress in raising awareness of substance misuse and formal teaching in this area to care of the elderly medicine services, social services, day centres and voluntary organisations.

A recent study of this service has shown that 40% of older people with dual diagnosis referred from medical in-patient settings achieved either abstinence or controlled drinking within the Mental Health of Older Adults Clinical Academic Group (Rao, 2013). Although there was no control group, this outcome is similar to outcomes in younger people.

The development of an integrated approach to care from a workforce that has competencies in the assessment and treatment of older people with substance misuse and co-occurring mental disorders remains the most pressing challenge for the 21st century.

Recovery can be a lengthy process and there are challenges, which include:

- Supporting accessing to specialist services (eg, overcoming stigma).
- Mobilising personal and social resources (eg, contact with family and friends, buddying and befriending, Alcoholics Anonymous attendance).
- Changing social contacts (eg, drinking partners).
- Achieving controlled drinking, rather than abstinence.
- Patient ownership of care plan.
- Emotional factors (eg, bereavement, loss, sexuality, histories of abuse, relationship problems, past traumatic experiences).
- Practical considerations (eg, presence of smoke alarm, diet, sleep, hazardous drug interactions, physical health, drinking and driving, trip hazards, safe storage of medications).
- Managing set-backs and not seeing them as failures.
- Managing harm reduction using a community reinforcement approach.

The most challenging problems for the recovery for older people with substance misuse in primary care is ARBD (Rao and Draper, 2015). The immediate management involve detoxification, general nutrition and the use of parenteral thiamine as necessary. Any co-morbidity or physical or psychological complications also need to be managed effectively, often in conjunction with mental health services. Longer-term management depends upon the person’s recovery during rehabilitation. Once ARBD is established, the prognosis for recovery can be split broadly into quarters: 25% make a complete recovery, 25% make a significant recovery, 25% make slight recovery and 25% make no recovery (MacRae and Cox, 2003).

If patients make a full or partial recovery they can be managed in the community with the help for the family and local services depending upon their disability. If they have made partial or no recovery during rehabilitation, then relocation to a care home may be the only viable option. ARBD presents a challenge to recovery in that older people with alcohol misuse and ARBD are less likely to move to controlled drinking or abstinence than those cognitively intact older people with alcohol misuse (Rao, 2013).

## The future of improving the quality of life for older people with substance misuse

Improving the health and social function of older people with substance misuse requires a range of interventions that are tailored the scale of the need.

These interventions are multi-faceted. A comprehensive assessment involves an age-sensitive approach that overcomes barriers such as stereotyping, stigma and therapeutic nihilism. Further research will be needed to inform more effective treatment. Training is essential for developing a workforce with the necessary knowledge, skills and attitudes. Clinical audit can improve service development through improving care pathways, particularly for older people with dual diagnosis; with an emphasis on quality and effectiveness. There is also a pressing need to develop closer links between primary care and the secondary care specialties of old age psychiatry, addictions and geriatric medicine. Lastly, the added value of working with carers and voluntary agencies cannot be underestimated.

It is impossible to prioritise all the above interventions, as they are integrally related. If staff are not adequately trained and supported, assessments will not be carried out sensitively and effectively; and the need for treatment will not be acknowledged. If policy does not steer professionals in the direction of age appropriate service developments, a focus on outcomes from better treatment implementation cannot become a reality. If funding is not secured, for research and audit, not only will future improvements will be stifled, but the costs of inaction are likely to exceed those of action.

The reality of significant change lies within primary care. Now, more than ever, there is the possibility of making a difference to the lives of older people with substance misuse.
